# Internal hernia to the retrosternal space is a rare complication after minimally invasive esophagectomy: three case reports

**DOI:** 10.1186/s40792-019-0578-9

**Published:** 2019-02-18

**Authors:** Takuji Sato, Takeo Fujita, Hisashi Fujiwara, Hiroyuki Daiko

**Affiliations:** Division of Esophageal Surgery, National Cancer Center Hospital East, 6-5-1 Kashiwanoha, Kashiwa, Chiba 277-8577 Japan

**Keywords:** Minimally invasive esophagectomy, Internal hernia, Retrosternal space, Retrosternal hernia

## Abstract

**Background:**

Minimally invasive esophagectomy is considered a beneficial approach to esophageal cancer, although a hiatal hernia occurs more frequently in this approach than in open esophagectomy with reconstruction via the mediastinal route. Development of an internal hernia to the retrosternal space is not a recognized complication of reconstruction via the retrosternal route after esophagectomy. We herein report three cases of the development of an internal hernia to the retrosternal space after minimally invasive esophagectomy.

**Case presentation:**

Thoracolaparoscopic esophagectomy with cervical anastomosis by retrosternal route reconstruction was performed in all three cases. All patients were men ranging in age from 60 to 80 years. Two patients had abdominal pain, and one had experienced syncope. All patients were diagnosed by computed tomography with an internal hernia to the retrosternal space and thoracic cavity (retrosternal hernia) without ischemic change to the incarcerated intestine. Two patients received medical therapy to relieve their intra-abdominal pressure, which allowed for a successful reduction of the intestine into the abdomen. Open laparotomy was performed to repair the hernia in the third patient. After reducing the intestine into the abdomen, reefing of the retrosternal orifice was performed, and the gastric conduit was anchored to the abdominal wall. No relapse occurred in three cases throughout follow-up.

**Conclusion:**

Hiatal hernia is a well-recognized complication after minimally invasive esophagectomy; however, retrosternal hernia is a rare complication following this procedure. Based on the present report, if no ischemic change is present in the herniated intestine, two types of potentially curative treatments are available: medical or surgical. As minimally invasive esophagectomy is performed more frequently, retrosternal hernia may become an increasingly more common complication in the near future.

## Background

The surgical approach to esophageal cancer is highly invasive and associated with high mortality and morbidity rates. However, minimally invasive esophagectomy (MIE) has been considered to reduce surgical injury compared with open esophagectomy (OE) [1]. A meta-analysis comparing MIE with OE demonstrated that MIE had short-term benefits. The estimated overall survival rate was also higher with MIE than OE. Therefore, MIE has become more common in the treatment of esophageal cancer and is considered a good method with which to reduce the high morbidity and mortality rates that are associated with OE [1].

Although MIE is considered beneficial, hiatal hernia (HH) occurs more frequently in MIE with reconstruction via the mediastinal route. A systematic review comparing HH after MIE versus OE showed that HH occurred more frequently after MIE (4.5%) than OE (1.0%) [2]. The retrosternal route is one reconstruction route that may be used after esophagectomy. Intrathoracic herniation of reconstructed organs was reported by Takayama et al. [3] and Uemura et al. [4], but no reports have described the incarceration of intestinal tissues other than reconstructed organs in the retrosternal space and thoracic cavity. We herein report three cases of internal hernia to the retrosternal space (hereafter called retrosternal hernia) after MIE.

## Case presentation

In 2010, the standard operation for esophageal cancer in our institution was thoracolaparoscopic esophagectomy (TLE) with three-field lymph node dissection using mediastinal route reconstruction and cervical esophagogastric anastomosis. After the development of HH, the reconstruction route was changed from the mediastinal to retrosternal route in 2014. The esophageal hiatus was completely closed with nonabsorbable sutures, and the retrosternal route was established using a laparoscopic technique. Unfortunately, we experienced three cases of retrosternal hernia.

**Fig. 1:**
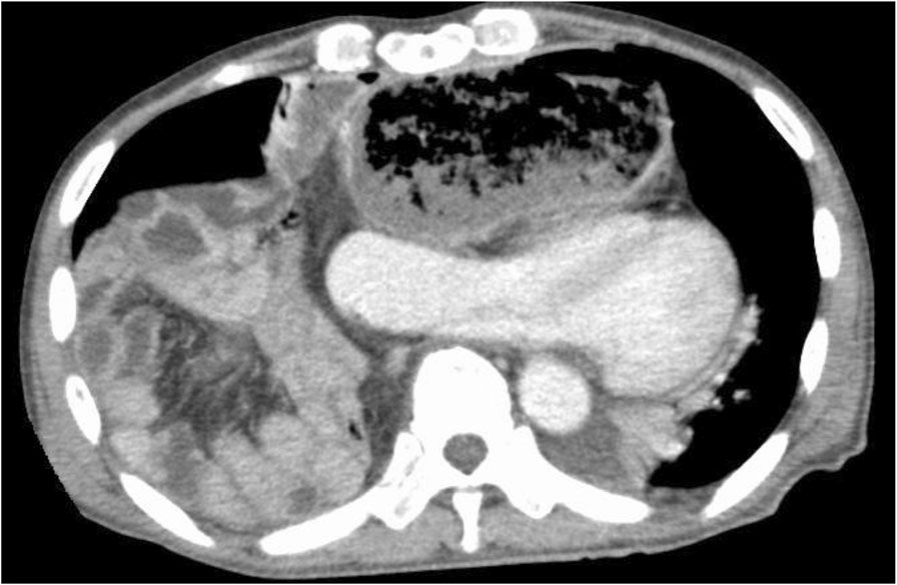
Computed tomography findings of case 1. The axial image demonstrated that the intestine had been drawn into both thoracic cavities. The intestine pressed the gastric conduit, leading to obstruction and dilation. This dilated gastric conduit severely pressed the heart

**Fig. 2:**
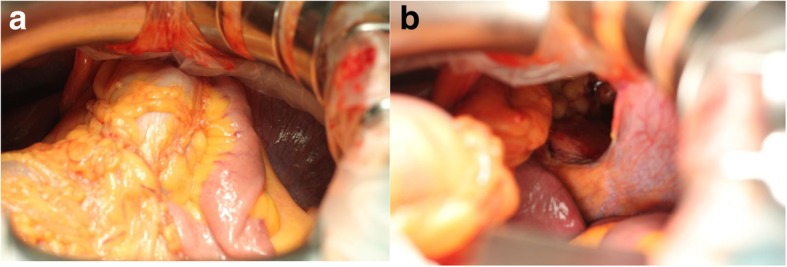
Intraoperative photographs of case 1. **a** The small and large intestines were incarcerated in the retrosternal orifice. **b** The intestine was easily reduced into the abdomen. The retrosternal orifice was widely opened with a sharp edge

Two of these three cases are herein described in detail. The first case involved a 74-year-old man with thoracic esophageal cancer. We performed TLE with retrosternal gastric conduit reconstruction. At 731 days after TLE, the patient experienced syncope and was referred to our hospital. Computed tomography (CT) showed that the small and large intestines were herniated into the bilateral thoracic cavities through the retrosternal space. The gastric conduit was obstructed by the herniated contents and was extremely dilated, severely pressing the heart (Fig. 1). We suspect that this severe heart pressure led to low blood pressure and resultant syncope. We diagnosed the patient with a retrosternal hernia with incarcerated intestine. The incarceration was repaired by open laparotomy. No adhesion was present in the abdomen or thoracic cavity. The herniated contents ran through the retrosternal orifice from the ventral aspect of the gastric conduit (Fig. 2a). The herniated contents were easily reducible and had no ischemic change. The orifice of the retrosternal space was widely opened, and the edge of the orifice had a burn injury (Fig. 2b). Reefing of the retrosternal orifice was performed with nonabsorbable sutures. Additionally, we anchored the gastric conduit to the ventral wall of the abdomen.

**Fig. 3:**
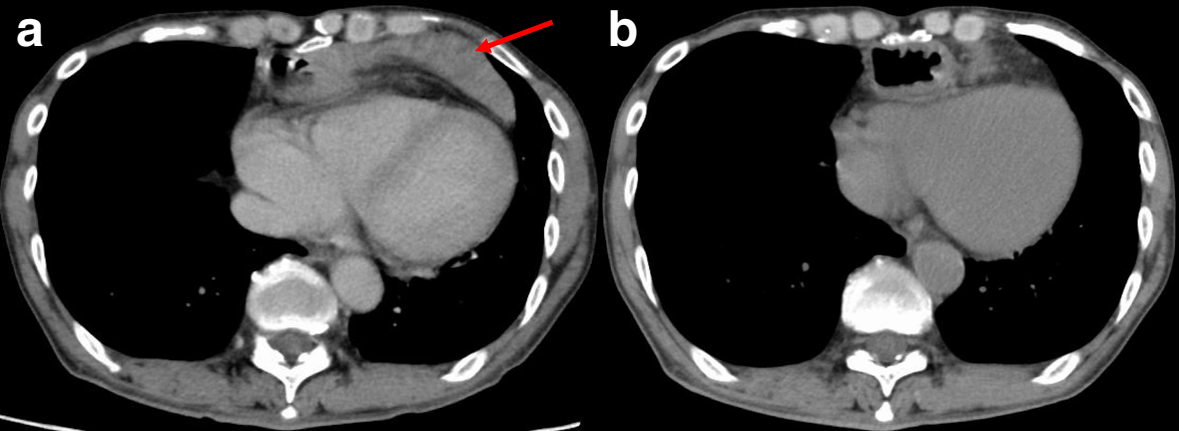
Computed tomography findings in case 2. **a** Computed tomography image obtained on the day of symptom onset. The intestine (red arrow) was dislocated only in the retrosternal space. **b** Computed tomography image obtained 2 days after symptom onset. The intestine had disappeared from the retrosternal space

The second case involved a 60-year-old man with thoracic esophageal cancer who underwent the same operation as in the first case. He developed sudden abdominal pain 436 days after TLE. CT showed that the small intestine was herniated into the retrosternal space without ischemic change (Fig. 3a). We treated him with an analgesic, which provided immediate pain relief without relapse. Two days later, no herniated intestine was present in the retrosternal space by CT (Fig. 3b).

From January 2014 to December 2016, retrosternal hernia occurred in 3 of 385 patients (0.007%) after TLE (Table 1). All patients were men with a body mass index (BMI) of < 20 kg/m^2^. The interval from TLE to diagnosis ranged from 413 to 731 days. The symptoms were sudden abdominal pain in two patients and syncope in one patient. Before the symptom onset, all patients were experiencing constipation. CT was useful for establishing the final diagnosis and showed that the herniated contents ran through the orifice to the retrosternal space and to the thoracic cavity from the ventral aspect of the gastric conduit in all patients. In addition, CT demonstrated that the herniated contents had no ischemic change in all patients.

**Table 1:** Cases of retrosternal hernia after thoracolaparoscopic esophagectomy

Case	Age (years)	Sex	BMI (kg/m^2^)	Symptom	Location of herniation	Diagnostic methods	Days until diagnosis	Herniated contents	Treatment	No relapse (months)
1	74	M	17.7	Syncope	Bilateral thorax	CT	731	SI/TC	Operation	15
2	60	M	18.2	Abdominal pain	Retrosternum	CT	485	SI	Analgesic drug	22
4	80	M	19.7	Abdominal pain	Left hemithorax	CT	413	SI/TC	Analgesic drug	15

*M* male, *BMI* body mass index, *CT* computed tomography, *SI* short intestine, *TC* transverse colon

Two patients were treated with analgesics to relieve pain and decrease the intra-abdominal pressure, which allowed the herniated contents to be reduced. However, one patient required surgical intervention, with no adhesions in either the abdomen or thoracic cavity. None of the three patients developed relapse throughout the follow-up period.

## Conclusions

MIE is being performed more frequently in patients with esophageal cancer, and a previous report indicated that HH occurs more often after MIE than after OE [2]. The reasons for this difference are the reduction of peritoneal adhesions in the hiatal region and the extensive dilation of the hiatus resulting from insufflation and iatrogenic manipulation during MIE [2, 5, 6]. Early ambulation as a part of an enhanced recovery program is also considered to contribute to the development of HH because it may increase the intra-abdominal pressure [5]. Additionally, some reports have described the relationship between BMI and HH [7, 8]. We previously reported the development of HH after OE [9] and MIE [10]. Unfortunately, we experienced three cases of retrosternal hernia after TLE.

A retrosternal hernia is one type of internal hernia defined as herniation of the intestine into the thoracic cavity by the retrosternal route. Retrosternal hernias may be categorized into two types: localized and extended. Localized hernias are located only in the retrosternal space. In contrast, extended hernias have spread not only to the retrosternal space but also to the thoracic cavity.

The common risk factors for both types of retrosternal hernia are the same as those for HH: reduction of peritoneal adhesions, extensive dilation of the retrosternal orifice, excessive intra-abdominal pressure, early mobilization, and low BMI. One risk factor that differs is injury to the mediastinal pleura induced by negative pressure derived from the thoracic cavity. Uemura et al. [4] concluded that injury to the mediastinal pleura introduces the reconstructed organ to the thoracic cavity by the negative pressure of breathing. We used the gastric tube as the reconstructed organ in the three patients described in the present report, but the intestine was drawn into the thoracic cavity by the retrosternal route. This is the first report to describe the herniation of the intestine instead of the reconstructed organs into the retrosternal space and thoracic cavity.

In our patients, no surgical injury to the pleura occurred during the laparoscopic procedure. However, even when no injury occurs during the operation, the mediastinal pleura may become weak because of the loss of surrounding tissue. This weak pleura may be intolerant to the high intra-abdominal pressure caused by constipation, leading to pleural injury. In addition, the retrosternal orifice was widely opened by a burn injury in one of our patients. This widely opened orifice might also lead to intestinal herniation.

As for treatment, the retrosternal hernia is controversial [2, 6, 11, 12]. Based on our experience, two approaches can be considered. First, if CT reveals no ischemic change to the herniated contents, reduction and control of the intra-abdominal pressure might lead to a reduction of the herniated contents into the abdominal cavity. In case 3, despite the presence of an extended type hernia with negative pressure derived from the thoracic cavity, relief of the intra-abdominal pressure successfully reduced the herniated contents. Second, if the decrease in the intra-abdominal pressure is unsuccessful, surgical repair is needed. We chose open laparotomy instead of laparoscopy because we expected postoperative adhesions around the retrosternal orifice. We also performed reefing of the dilated retrosternal orifice and anchored the gastric conduit to the ventral wall. This procedure may be essential for preventing herniation after MIE. If this suturing technique is enough to prevent a retrosternal hernia, then laparoscopic repair is another treatment choice.

To prevent a retrosternal hernia after MIE, close attention must be paid to the procedure by which the retrosternal route is established. Uemura et al. [4] concluded that the use of a video-assisted maneuver might prevent injury to the parietal pleura during blunt dissection of the retrosternal space. Based on our cases, the retrosternal orifice should not be opened widely. The retrosternal orifice should be of adequate size for the gastric conduit and omentum with respect to both vascularity and hernia formation. A small amount of the omentum and a large orifice will lead to a retrosternal hernia. The pleura should not be injured without dissection of the surrounding tissue, and the intra-abdominal pressure must be controlled.

In summary, we experienced three cases of a retrosternal hernia as a rare complication after MIE. Surgeons should be aware of this uncommon complication if retrosternal route reconstruction is used after MIE. As MIE is performed more frequently in the near future, retrosternal hernia may become an increasingly more common complication.

## Abbreviations

BMI: Body mass index
CT: Computed tomography
HH: Hiatal hernia
MIE: Minimally invasive esophagectomy
OE: Open esophagectomy
TLE: Thoracolaparoscopic esophagectomy
